# Compressive Properties and Energy Absorption Behavior of 316L Steel Foam Prepared by Space Holder Technique

**DOI:** 10.3390/ma16041419

**Published:** 2023-02-08

**Authors:** Guangyu Hu, Guili Xu, Qiang Gao, Zhanhao Feng, Peng Huang, Guoyin Zu

**Affiliations:** School of Materials Science and Engineering, Northeastern University, Shenyang 110819, China

**Keywords:** steel foam, porosity, pore size, quasi-static compression properties, energy absorption, 316L stainless steel, space holder technique

## Abstract

The effect of porosity and pore size on the quasi-static compression properties and energy absorption characteristics of the steel foam was investigated in this paper. The 316L steel foams were prepared through powder metallurgy using urea as the space holder. The macrostructure of steel foam and microstructure of the pore walls were characterized, and the quasi-static compression experiments were conducted on the specimens in the axial direction at a strain rate of 10^−3^ s^−1^. The results show that the increase in porosity decreases the yield strength and plastic modulus of the steel foam but increases the densification strain of the steel foam. The yield strength of the steel foam decreases significantly when the pore size is 2.37 mm. However, the pore size has little effect on the plastic modulus. Moreover, the energy absorption per volume of the steel foam decreases with increasing porosity at the same strain. The effect of porosity on energy absorption efficiency is greater than that of pore size.

## 1. Introduction

Metal foam has many excellent properties, such as low density, high specific strength, high specific energy absorption capacity, favorable sound absorption, and electromagnetic shielding [1,2,3]. Steel foam has higher toughness, superior impact absorption, high-temperature service performance, and excellent corrosion resistance compared with widely applied aluminum foam [4,5]. Therefore, steel foam is expected to be a commonly used material in construction, vehicles, ships, bio-implant, and defense [6,7].

Currently, steel foam can be prepared by investment casting method [8], hollow sphere sintering method [9], space holder technique [10], etc. The investment casting method is a complex production process, and it is difficult for molten liquid metal to flow into the interstices of the filler particles. The preparation of metal hollow spheres is more complicated in the hollow sphere sintering method. The space holder technique is a feasible, simple-operated, and cost-controlled technology for preparing steel foam. The porosity, pore size, and pore shape can be controlled by the amount, size, and shape of the space holder, which cannot be achieved by other processes [11].

Many space holders can be used for the preparation of steel foam, such as CaCl_2_ [12], NH_4_HCO_3_ [13], NaCl [14], starch [15], sodium thiosulfate [16], etc. Compared to other space holders, urea is easy to obtain at a low cost, and its shape and size can be controlled. Meanwhile, urea can be entirely removed by evaporation, which is easy to achieve. Therefore, urea is favored by researchers as a space holder for preparing steel foam. Jain et al. [17,18,19,20] prepared steel foam materials with 55~81% porosity using urea as a space holder. The compressive properties of the steel foam were investigated for different relative densities, different steel powder microstructures, and different compressive strain rates. Sazegaran et al. [21] used urea as the space holder to prepare the steel foam. The influence of preparation process parameters such as forming pressure, sintering temperature, and sintering time on the compression energy absorption characteristics was investigated. Mondal et al. [13] prepared 316 stainless steel foams with different porosity by powder metallurgy using NH4HCO3 as a space holder and investigated the compressive properties at sintering temperatures of 1100 °C and 1200 °C. Nuray et al. [22] investigated the structural and mechanical properties of low alloy steel foams variations resulting from the use of spacer particles having different amounts and sizes. In the above studies, the main focus is on the effect of pore structure and preparation parameters on the mechanical properties of steel foam. In addition, the analysis and research on the energy absorption characteristics of metal foams mainly focus on aluminum foams [23,24,25], while there are few reports on the energy absorption of steel foams. It is necessary to study pore structure for energy absorption of steel foam.

In this study, steel foam with different porosity and pore size was prepared by powder metallurgical route using urea as the space holder and 316L stainless steel powder as the matrix. The effects of porosity and pore size on the compressive stress-strain curve, yield strength, elastic modulus, plastic modulus, and energy absorption efficiency of the steel foams were analyzed and discussed. To choose different pore structures under different application conditions.

## 2. Materials and Methods

### 2.1. Raw Materials

**Figure 1:**
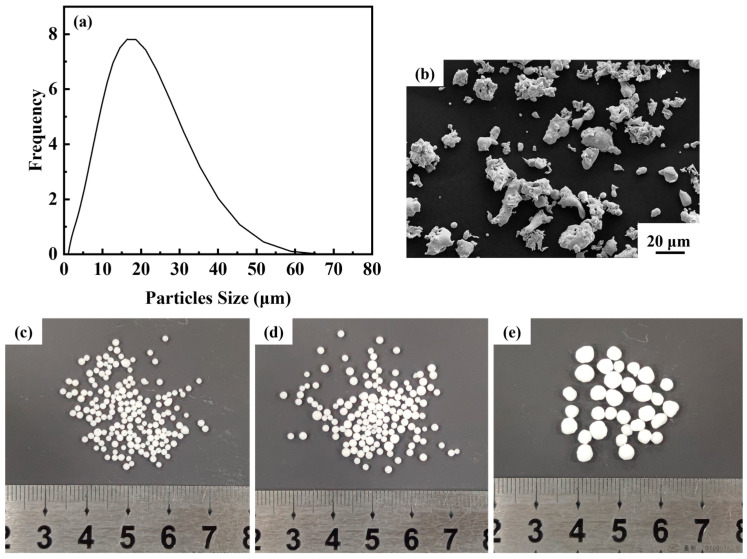
(**a**) Particle size distribution of steel powder; (**b**) microscopic morphology of steel powder; (**c**–**e**) urea particles of different size ranges.

The matrix metal powder is austenitic stainless steel 316L powder supplied by Handan Esier Atomizing Powder Co., Ltd. (Handan, China) and the space holder is urea particles provided by Shandong Luyang Eco-Fertilizer Co., Ltd. (Zibo, China) Urea particles with sizes ranging from 0.9 mm to 1.6 mm, 1.6 mm to 2.5 mm and 2.5 mm to 4.5 mm were screened using standard test sieves. The morphological characterization of 316L powders was observed using scanning electron microscopy (Zeiss ULTRA PLUS, Oberkochen, Germany). The powders were also examined for particle size distribution using a laser diffraction particle analyzer (Mastersizer 3000, Malvern Panalytical, Malvern, UK). The particle size distribution and microscopic morphology of 316L powders, and the macroscopic morphology of urea are shown in Figure 1. Figure 1a illustrates that 90% of particle size is below 32.3 μm and the average particle size is 15.5 μm. The microscopic morphology of the steel powder is irregular (Figure 1b), and the urea particles are in the shape of spheres (Figure 1c–e).

### 2.2. Preparation Process

**Figure 2:**
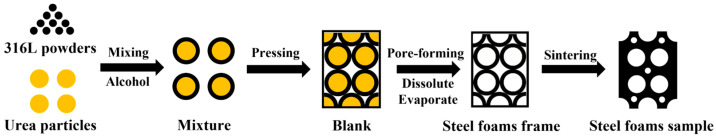
Process flow diagram for making steel foam.

The preparation process is divided into four main stages: (1) mixing, (2) pressing, (3) pore-forming, and (4) sintering. The process flow diagram for making steel foams is shown in Figure 2.

Firstly, the 316L stainless steel powders were mixed with urea particles in different volume fractions. Alcohol, about 4~6% of the total mass of the mixed powder, was added as a binder. As shown in Equation (1), the mass fraction ratio required for mixing at different porosities is calculated [13].

(1) $$\frac{m_{s}}{m_{u}}=\frac{\rho_{s}(1-P_{t})}{\rho_{u}P_{t}}$$

where *m*_s_ denotes the mass of 316L steel powder, *m*_u_ denotes the mass of urea, *ρ*_s_ denotes the density of 316L steel (7.98 g/cm^3^), *ρ*_u_ is the density of urea (1.335 g/cm^3^), *P*_t_ is the target porosity of the steel foam. The mixed powders were left in a three-dimensional mixer for 30 min to ensure that the steel powders were evenly coated on the surface of the urea particles. Secondly, the powder mixture was pressed at room temperature using a hydraulic press with a pressing stress of 300 MPa and a holding time of 5 min. The size of the pressed blank after molding was 30 mm in diameter and 25 ± 2 mm in height. The pressed blank was soaked in cold water for 1 h to dissolve most of the urea particles and then dried in a blast drying oven at 120 °C for 1 h. Then, the pressed blank was held at 450 °C for 1 h in the air to remove the urea remaining inside the pressed blank to obtain the steel foam frame. Finally, the pressed frame was sintered at 1200 °C, holding for 2 h in a vacuum (0.1 Pa), and the steel foam samples were obtained by cooling with the furnace.

### 2.3. Microstructure Characterization and Mechanical Property Characterization

The steel foams were cut into cylindrical shapes of Φ20 × 20 mm for porosity measurement and mechanical property testing. The actual porosity of the steel foam samples was calculated using the mass-volume method [21] as shown in Equation (2).

(2) $$P_{a}=(1-\frac{m}{V\rho_{s}})\times 100\%$$

where *P*_a_ is the actual porosity of the steel foam. *m* and *V* are the actual measured mass and volume of the steel foam, respectively. The values of relative density and actual porosity sum to 1.

The steel foams were cut into 10 × 10 × 10 mm cubes, the surface was polished to bright, and the longitudinal section of the sample (i.e., the interface parallel to the pressing direction) was scanned using a scanner. The average pore size and roundness of the longitudinal section of the sample were counted by Image-pro-plus software v6.0. The sintered steel foam samples were polished using a standard metallographic polishing technique and etched using chemical reagent (5g FeCl3 and 50 mL HCl). The microstructure of the samples was observed and analyzed using scanning electron microscopy (Zeiss ULTRA PLUS-15kV-SE2). The quasi-static compression performance of the steel foam samples was tested at room temperature using a universal testing machine (AG-XPLUS100KN, Shimadzu, Kyoto, Japan) with a beam movement rate of 1.2 mm/min. For each experimental condition, at least three samples were tested.

The energy absorption characteristics of steel foam were analyzed by energy absorption (*W*), energy absorption efficiency (*E*), and ideal energy absorption efficiency (*I*). Equation (3) is calculated as the absorbed energy value per unit volume.

(3) $$W=\int_{0}^{\varepsilon_{m}}\sigma d\varepsilon$$

where *W* is the energy absorption per unit volume, *ε_m_* is the arbitrary strain, *σ* is the stress, *ε* is the strain, and *σ* is a function of *ε*.

The determination of steel foams’ optimal energy absorption condition can be done by using the energy absorption efficiency parameter. The ideal energy absorption efficiency reflects the closeness of the foam steel to the ideal energy-absorbing material.

(4) $$E=\frac{\int_{0}^{\varepsilon_{m}}\sigma d\varepsilon }{\sigma_{m}}$$

(5) $$I=\frac{\int_{0}^{\varepsilon_{m}}\sigma d\varepsilon }{\sigma_{m}\varepsilon_{m}}$$

where *E* is the energy absorption efficiency, which indicates the energy absorption efficiency corresponding to the flow stress of the steel foam in the process of compression deformation. *I* is the ideal energy absorption efficiency, that is, the ratio of the area enclosed by the stress-strain curve of the steel foam at any strain to the rectangular area in which it is located.

## 3. Results and Discussion

### 3.1. Macrostructure and Microstructure

It is assumed that the pores of the steel foam are only created by the release of the urea. Then, different target porosity can be obtained by controlling the amount of urea added (i.e., the volume fraction of urea to the mixed powders) and the pore size by controlling the size of the urea.

**Figure 3:**
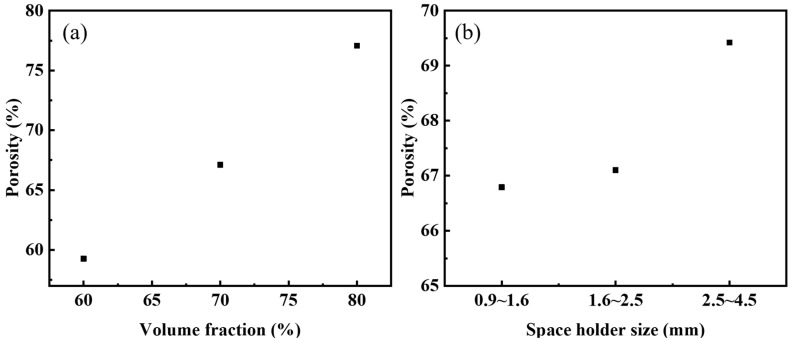
Actual porosity versus (**a**) volume fraction of urea and (**b**) size of urea.

The correspondence between the volume fraction of urea (target porosity) and the actual porosity of the steel foams is shown in Figure 3a. The actual porosity is lower than the target porosity as seen in the figure. The volume of the sample is reduced due to the diffusion bonding of the metal particles during the sintering process, which increases its density and reduces porosity [26]. When the volume fraction of the added urea is 70%, the relationship between the size of the urea and the actual porosity of the steel foams is shown in Figure 3b. As seen in the figure, the actual porosity of all three samples is less than 70%. When the urea size is 2.5~4.5 mm, its porosity is closest to the target porosity. The role of urea in the preparation process is not only as a space holder, but urea is organic and acts as a lubricant in the powder pressing process. Therefore, the powder mixture’s compressibility becomes higher when the amount of urea added increases, leading to a higher density of the pressed blank and thus enhancing the sintering ability. Thus, the difference between the target and actual porosity is greater when the urea content is 80% than 60%. Changing the particle size of the urea will change the average pore size of the steel foams and the porosity, but the effect on the porosity is small. Table 1 shows the actual porosity, average pore size, and pore roundness. When the particle size of urea is 2.5–4.5 mm and 0.9–1.6 mm, the average pore size of the steel foam longitudinal section is 2.37 mm and 1.04 mm, and the porosity is 69.42% and 66.79%, respectively. The reduction in pore size is accompanied by a slight reduction in porosity.

**Figure 4:**
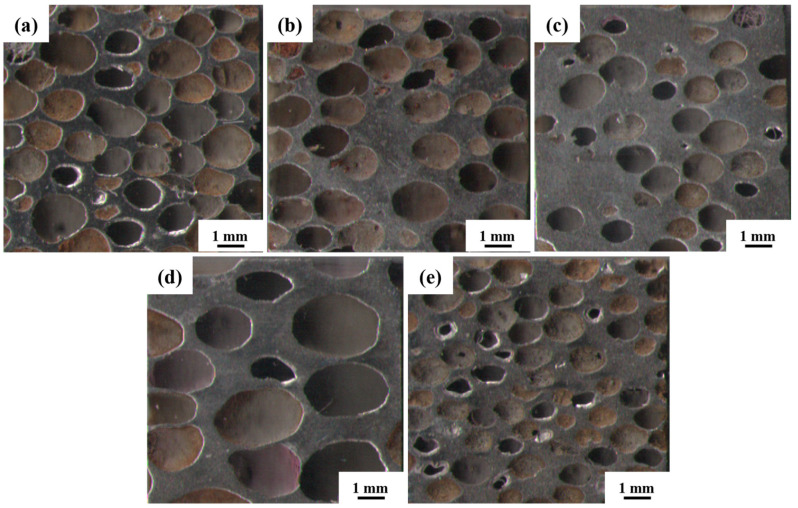
Macroscopic morphology of the longitudinal section of (**a**) 1#, (**b**) 2#, (**c**)3#, (**d**) 4#, (**e**) 5# samples.

The macroscopic morphology of the steel foam is shown in Figure 4. As shown in Figure 4, as the porosity increases or the pore size decreases, the distribution of macroscopic pores is more uniform, and the linkage between pores is more prominent. As seen in Table 1, the pore size of all five samples is smaller than the size of the urea, which is caused by the shrinkage of the steel foam during the sintering process and is also related to the squeezing and deformation of the urea during the pressing process [27]. When the porosity is 77.07% and 59.27%, the corresponding pore size is 1.58 mm and 1.10 mm, respectively. The reason for this phenomenon may be related to the processing property of the powder mixture. This phenomenon is similar to the study results of Xiao et al. [28]. When the added urea is 80%, the pore roundness is 1.35 and the deformation of the pore is more extensive. During the pressing of the powder mixture, the pressure on the urea will also be greater when the urea addition is higher. That would result in the pore shape of the steel foam being pressed into an oval shape from spherical. The pore roundness is 1.52 when the size of the added urea is 2.5~4.5 mm. This is because the size of urea increases and the distribution of the pores is less uniform when the amount of urea added is certain. During the pressing process, some pores are subjected to larger forces, resulting in larger deformations.

**Figure 5:**
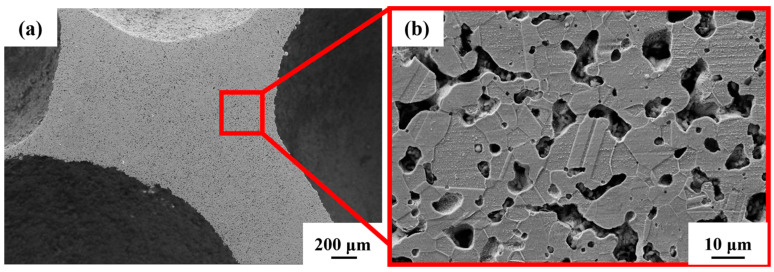
Microstructure of (**a**) steel foam and (**b**) pore wall.

From Figure 5a, the existence of pores in the steel foam is divided into two forms. One is the macroscopic macropores left after removal by urea, and the other is the micropores on the pore wall produced by the sintered shrinkage of the steel matrix powder. Previous studies, whether using metal magnesium, spherical urea, or sodium chloride as space holders, have also shown that the prepared metal foam contains pores in two scales [29,30,31]. Due to the incomplete sintering of steel powder in the sintering process, the pore wall of the sintered sample contains micron-scale pores (Figure 5b), whose size is between 1–10 μm. In Figure 5b the grain boundaries and austenitic twin morphology formed after the sintering of the particles can be clearly seen.

### 3.2. Compression Mechanical Properties

**Figure 6:**
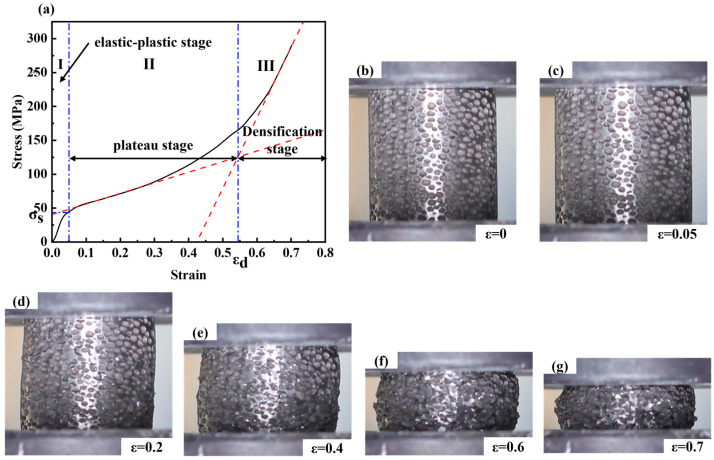
(**a**) Stress-Strain curve of the steel foam, and the section image with different deformation: (**b**) ε = 0; (**c**) ε = 0.05; (**d**) ε = 0.2; (**e**) ε = 0.4; (**f**) ε = 0.6; (**g**) ε = 0.7; (The red lines in (**a**) are linear fits for strains 0.1 to 0.3 and 0.65 to 0.70, respectively. The blue lines in (**a**) correspond to strain 0.05 and densification strain respectively).

As known from Figure 6, the compression deformation process can be divided into three deformation stages under an external load with a strain rate of 0.001 s^−1^ (i.e., a beam speed of 1.2 mm/min and a scale distance of 20 mm).

The first stage is the elastic-plastic stage, where the stress increases linearly and the pore wall tends to rupture and bend with strain. Steel foam does not have a completely absolute area of elastic deformation, which is very different from ordinary solid materials. The non-uniformity of the pore walls, local density, pore size, and pore roundness of the steel foams leads to the yielding of individual pore walls. Therefore, it is difficult to determine the exact value of the elastic modulus here. The second stage is the plastic-yielding stage, also known as the plateau stage, in which the pore wall collapses due to excessive buckling, followed by layer-by-layer shearing and rupture. At the plateau stage, the stress increases with the increase of strain, and higher porosity will make the curve flatter at this stage. Here, the stress corresponding to a strain of 0.05 is called the yield strength [23]. A linear fit to the stress-strain curve of the plateau stage is performed and the slope of the obtained slope line is the plastic modulus. The third stage is the densification stage, in which the steel foams become a solid material and the stress increases very much with strain during this stage. The densification strain is obtained as the strain corresponding to the point where the plateau and densification stages intersect after a linear fit [32]. The densification strain is an essential indicator of the energy absorption capacity of the metal foams. It is the cut-off point for the transition of the metal foams from the plateau stage to the densification stage. During the compression of the sample, the cross-sectional area of the sample changes almost constantly in the elastic-plastic stage and plateau stage and increases with the decrease of the sample height in the densification stage.

**Figure 7:**
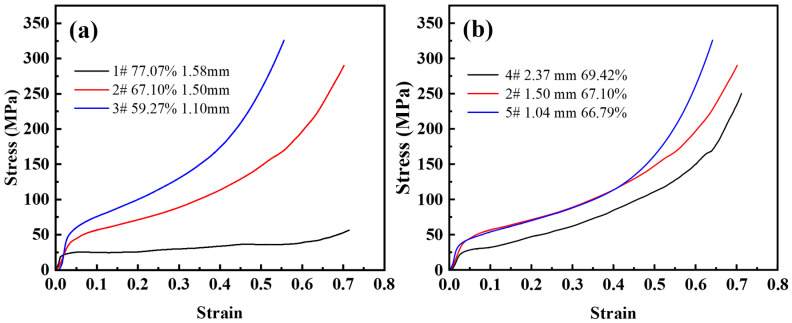
Compressive stress-strain curve of (**a**) different porosity and (**b**) different pore size.

Figure 7a shows the compressive stress-strain curves for the urea size of 1.6~2.5 mm and the volume fraction of urea additions in the range of 60–80%. Under axial unidirectional load, pore wall bending, collapsing, and finally yields a smaller compressive strain. If the stress in the pore walls exceeds the yield stress of the matrix material, plastic deformation begins and the deformation is irrecoverable. Therefore, the elastic modulus of the steel foam is difficult to determine. As the porosity decreases, it is obvious that the effect of strain hardening becomes more visible, the plateau stage ends earlier and enters the densification stage earlier. From Table 2, the corresponding densification strains were 0.43, 0.54, and 0.65 when the porosity of the samples was 59.27%, 67.10%, and 77.07%, respectively. It can be seen that the curve is smoother when the porosity is low. When the porosity is high, the stress-strain curves of the steel foam samples start to fluctuate during the plateau stage. The yield strength also increases significantly with the decrease in porosity. This is because the higher the porosity, the greater the number of pores, the thinner the pore walls, and the lower the stresses that can be withstood.

Figure 7b shows a set of compressive stress-strain curves for the same amount of urea addition and different urea sizes. It can be seen from the figure that the plateau stage is almost the same, and the elastic modulus varies slightly. In the plateau stage, the yield strength of the sample with a larger pore size is significantly lower than that of the sample with a smaller pore size. The strain-hardening effect is almost the same for the three samples. The elastic-plastic and plateau stages of the curves with pore sizes of 1.50 mm (sample 2) and 1.04 mm (sample 5) almost overlap, and the compressive strength of the smallest pore size is greater in the densification phase. When the pore size of the steel foam is adjusted by changing the size of the urea, the porosity also increases with the increase of the urea size, which is the possible cause of this phenomenon.

Table 3 lists the pore structure and the mechanical properties of steel foam. It should be noted that the stress-strain curves of steel foam with different matrices are very different. The almost linear initial loading curve of the 316L steel foam is extended by a hardening plateau with a slightly increasing stress level. In the case of the 17-4 PH steel foams, a stress peak is observed after a nearly linear elastic regime. This is followed by a sudden decrease in stress [2,21,22,33]. This will make a difference in the way the characteristic quantity of compression performs.

### 3.3. Energy Absorption Characteristics

Energy absorption refers to the energy absorbed per unit volume of the specimen when compressed to a certain strain, geometrically expressed as the area contained under the stress-strain curve. It is an important characteristic value to measure whether the metal foams can have good energy absorption capacity during compression deformation.

**Figure 8:**
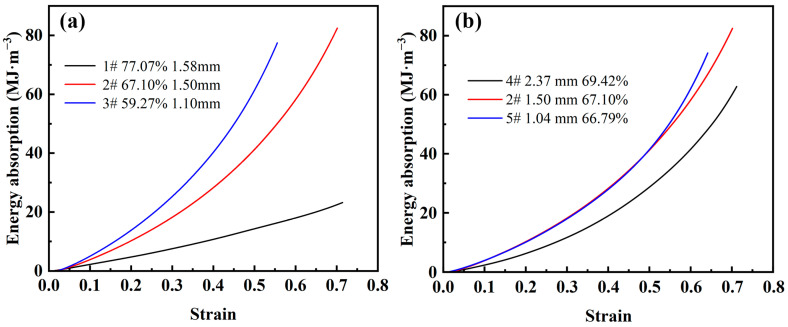
Energy absorption-strain curve of steel foam of (**a**) different porosity and (**b**) different pore size.

Figure 8a shows the energy absorption-strain curves for different porosities. As can be seen from the figure, the energy absorption value of the steel foam is smaller in the elastic-plastic stage. This is because the energy absorption of steel foams mainly relies on the collapse of the pore structure to absorb energy, mainly in the plateau stage. Also, it can be found that the energy absorption value increases linearly with the increase of strain at higher porosity. As the porosity decreases, the energy absorption value shows a power function increase with the increase of strain. This is caused by the significant strain-hardening effect of the steel foam and the elevated plastic modulus when the porosity is low. When the steel foam reaches the densification strain, the energy absorption value increases extremely with the increase of stress.

Figure 8b shows the energy absorption-stress curves for different pore sizes. As can be seen from the figure, the energy absorption of the larger pore size is always lower than that of the steel foam with smaller pore size. The curves of the two samples with smaller pore sizes almost overlap, and the sample with the smallest pore size absorbs the largest energy value after reaching the densification strain. When the pore size is 2.37 mm (sample 4), the yield strength decreases significantly, and the plastic modulus and densification strain do not change much, which is the main reason that affects the energy absorption energy value. This shows that steel foam of a small pore size has better energy absorption performance.

### 3.4. Energy Absorption Efficiency

To further analyze the energy absorption capacity, Miltz et al. [35] proposed two concepts of energy absorption efficiency and ideal energy absorption efficiency to evaluate the energy absorption properties of metal foam materials.

**Figure 9:**
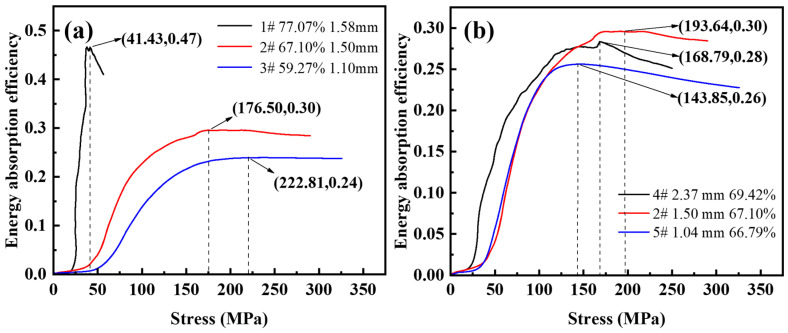
Energy absorption efficiency-stress curve of (**a**) different porosity and (**b**) different pore size.

Figure 9 shows the energy absorption efficiency-stress diagram for different porosity and pore sizes. As shown in Figure 9a, the maximum energy absorption efficiency values with a porosity of 59.27~77.07% are 0.24, 0.30, and 0.47. For steel foam with high porosity, the energy absorption efficiency peaks as the stress increases during compression deformation, and then the energy absorption efficiency decreases as the stress increases. For the curves with a porosity of 59.27~67.10%, when the energy absorption efficiency reaches its peak, the energy absorption efficiency remains almost stable and does not decrease with the increase of stress. This is because the thicker the pore wall, the higher the plastic modulus in the plateau stage as the porosity decreases, and the more visible the strain-hardening effect. The minimum stresses corresponding to the peak energy absorption efficiency are 222.81 MPa, 176.50 MPa, and 41.43 MPa. This stress value is approximately the same as the stress value corresponding to the densification strain on the stress-strain curve. It indicates that the steel foam can fully exploit its energy absorption properties when the pores are fully compacted.

As shown in Figure 9b, the maximum energy absorption efficiencies from small to large pores were 0.26, 0.30, and 0.28, and the peak energy absorption efficiency corresponds to stresses of 143.85 MPa, 193.64 MPa, and 168.79 MPa, respectively. It can be seen that the energy absorption efficiency of the steel foam is not monotonically varying with the pore size. The highest energy absorption efficiency is achieved when the size of the urea is 1.6~2.5 mm, the pore size of the longitudinal section is 1.50 mm (sample 2), and the corresponding peak stress is the largest.

**Figure 10:**
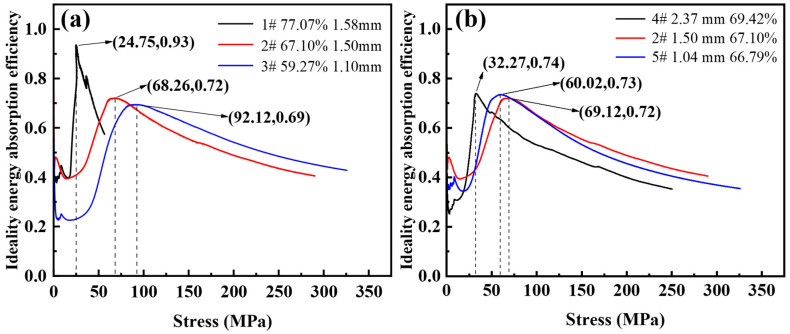
Ideal energy absorption efficiency-stress curve of (**a**) different porosity and (**b**) different pore size.

Figure 10 shows the ideal energy absorption efficiency-stress curves with different porosity and pore sizes. As shown in Figure 10a, the corresponding maximum ideal energy absorption efficiencies are 0.69, 0.72, and 0.93 with a porosity of 59.27~77.07%. The stress values for the peak ideal energy absorption efficiencies are 92.12 MPa, 68.26 MPa, and 24.75 MPa, respectively. It can be seen that when the porosity is higher, the ideal energy absorption efficiency is closer to 1. This is because the higher the porosity, the smaller the plastic modulus at the plateau stage, and the steel foam is closer to the ideal energy-absorbing material during compression deformation. The efficiency of the foam is influenced by its hardening behavior. The energy absorption capacity increases with increasing strain hardening while the energy absorption efficiency decreases [36].

As shown in Figure 10b, the corresponding maximum ideal energy absorption efficiencies are 0.73, 0.72, 0.74, and the stresses corresponding to the peak ideal energy absorption efficiencies are 60.02 MPa, 69.12 MPa, 32.27 MPa with the pore sizes from 1.04 mm to 2.37 mm, respectively. It can be seen that the ideal energy absorption efficiency of the three samples is almost the same, and the pore size factor has little effect on the ideal energy absorption efficiency of the steel foam. This is because the plastic modulus of the three samples is 172.68 MPa, 151.31 MPa, and 152.01 MPa, and the plastic modulus is almost the same.

## 4. Conclusions

Steel foams were prepared through powder metallurgy using urea as the space holder and 316L stainless steel powder as matrix material in this research work. The influences of the porosity and pore size on porosity, the morphology of the pores, the microstructure of the pore walls, compressional properties, and energy absorption properties were studied by a series of designated experiments. The following remarks can be concluded:

1. The actual porosity of the steel foam is lower than the target porosity under the condition of vacuum sintering at 1200 °C for 2 h, and the pore size is lower than the size of urea.
2. Porosity has a significant effect on the compressive deformation behavior of the steel foam prepared by powder metallurgy. A larger pore size leads to an increase in porosity and a decrease in its mechanical properties.
3. In the plastic deformation zone of the steel foams, the strain hardening effect is significant, the plastic modulus is more sensitive to the variation of porosity, and the variation of pore size has little impact on it.
4. The increase in porosity increases the energy absorption per volume, energy absorption efficiency, and ideal energy absorption efficiency. The effect of the change of pore size on the energy absorption efficiency and ideal energy absorption efficiency is not particularly significant.

There are many properties worth studying in steel foam. In practical applications, the load on steel foam is very complicated. There are many contents worth studying, such as high-speed deformation properties, high-temperature mechanical properties, etc. The steel foam prepared by the space holder technique has anisotropy of pore shape, and the compression properties in different directions will be studied later. And it is necessary to make the steel foam pore shape more regular by improving the process.

## Figures and Tables

**Table 1:** Statistics of pore structure parameters.

Sample Number	Target Porosity (%)	Space Holder Size (mm)	Actual Density (g/cm^3^)	Relative Density	Actual Porosity (%)	Average Pore Size (mm)	Average Pore Roundness
1#	80	1.6~2.5	1.83 ± 0.01	0.23 ± 0.01	77.07 ± 0.01	1.58 ± 0.26	1.35 ± 0.06
2#	70	1.6~2.5	2.63 ± 0.01	0.33 ± 0.01	67.10 ± 0.01	1.50 ± 0.32	1.25 ± 0.07
3#	60	1.6~2.5	3.25 ± 0.01	0.41 ± 0.01	59.27 ± 0.01	1.10 ± 0.24	1.25 ± 0.04
4#	70	2.5~4.5	2.44 ± 0.01	0.31 ± 0.01	69.42 ± 0.01	2.37 ± 0.48	1.52 ± 0.05
5#	70	0.9~1.6	2.65 ± 0.01	0.33 ± 0.01	66.79 ± 0.01	1.04 ± 0.20	1.27 ± 0.05

**Table 2:** Mechanical properties of steel foam.

Sample	Yield Strength (MPa)	Elastic Modulus (GPa)	Plastic Modulus (MPa)	Densification Strain
1#	25.25 ± 0.37	2.54 ± 0.55	29.62 ± 0.25	0.65 ± 0.03
2#	45.21 ± 0.32	1.97 ± 0.51	151.31 ± 0.24	0.54 ± 0.03
3#	60.14 ± 0.30	3.48 ± 0.49	281.54 ± 0.27	0.43 ± 0.03
4#	28.39 ± 0.36	1.49 ± 0.54	152.01 ± 0.30	0.60 ± 0.03
5#	44.04 ± 0.29	2.83 ± 0.47	172.68 ± 0.19	0.52 ± 0.03

**Table 3:** Comparison of properties of steel foam under quasi-static loading.

Metal Matrix	Porosity (%)	Pore Size (mm)	Yield Stress (MPa)	Densification Strain	Ref
Fe-0.6C-2P	75–80	1.2–1.6	15–45		[21]
316L	56–81	1.57–1.89	5.4–45.3	0.32–0.43	[20]
316L	54–80	1.65–1.95	5.95–30.73	0.38–0.62	[18]
316-L45	37.58–61.84	2	50–90		[34]
17-4 PH	40–80	0.45–0.92	50–80		[2]
Fe–1.75Ni–1.5Cu–0.5Mo–0.6C	47.8–70.9	0.38–0.10	17–116		[22]
17-4 PH	40–80	0.45–0.92	60–290		[33]
316L	59.27–77.07	1.04–2.37	25.25–60.14	0.43–0.65	This paper

## Data Availability

Not applicable.
